# The Application of Resveratrol Derivatives in Oral Cells Reduces the Oxidative Stress Induced by Glucocorticoids

**DOI:** 10.3390/metabo14070350

**Published:** 2024-06-22

**Authors:** Emira D’Amico, Chiara Cinquini, Morena Petrini, Antonio Barone, Giovanna Iezzi, Simonetta D’Ercole, Barbara De Filippis, Tania Vanessa Pierfelice

**Affiliations:** 1 Department of Medical, Oral and Biotechnological Sciences, University “G. d’Annunzio” of Chieti-Pescara, Via dei Vestini 31, 66100 Chieti, Italy; emira.damico@unich.it (E.D.); gio.iezzi@unich.it (G.I.); simonetta.dercole@unich.it (S.D.); tania.pierfelice@unich.it (T.V.P.); 2 Department of Surgical, Medical, Molecular Pathologies and of the Critical Needs, School of Dentistry, University of Pisa, 56126 Pisa, Italy; chiara.cinquini@gmail.com (C.C.); antonio.barone@unipi.it (A.B.); 3 Complex Unit of Stomatology and Oral Surgery, University-Hospital of Pisa, 56126 Pisa, Italy; 4 Department of Pharmacy, “G. d’Annunzio” University of Chieti-Pescara, Via dei Vestini 31, 66100 Chieti, Italy; barbara.defilippis@unich.it

**Keywords:** resveratrol derivatives, sulfonamides, oxidative stress, oral cells, glucocorticoids

## Abstract

Oxidative stress and high levels of reactive oxygen species (ROS) are linked to various age-related diseases and chronic conditions, including damage to oral tissues. Dexamethasone (DEX), a widely used glucocorticoid in dentistry, can have side effects like increased ROS production and delayed wound healing. Resveratrol (RSV) is known for its antioxidant properties, but its limited bioavailability hinders its clinical use. This study investigated the potential of two RSV derivatives (**1d** and **1h**) to address these limitations. The antioxidant abilities of **1d** and **1h** (5 μM) against DEX-induced oxidative stress (200 μM) were evaluated in human gingival fibroblasts (hGFs) and osteoblasts (hOBs). The effects of these compounds on cell viability, morphology, ROS levels, SOD activity, gene expression, and collagen production were evaluated. RSV derivatives, under DEX-induced oxidative stress condition, improved cell growth at 72 h (191.70 ± 10.92% for **1d**+DEX and 184.80 ± 13.87% for **1h**+DEX), morphology, and SOD activity (77.33 ± 3.35 OD for **1d**+DEX; 76.87 ± 3.59 OD for **1h**+DEX at 1 h), while reducing ROS levels (2417.33 ± 345.49 RFU for **1d**+DEX and 1843.00 ± 98.53 RFU at 4 h), especially in hOBs. The co-treatment of RSV or derivatives with DEX restored the expression of genes that were downregulated by DEX, such as HO-1 (1.76 ± 0.05 for **1d**+DEX and 1.79 ± 0.01 for **1h**+DEX), CAT (0.97 ± 0.06 for **1d**+DEX and 0.99 ± 0.03 for **1h**+DEX), NRF2 (1.62 ± 0.04 for **1d**+DEX and 1.91 ± 0.05 for **1h**+DEX), SOD1 (1.63 ± 0.15 for **1d**+DEX and 1.69 ± 0.04 for **1h**+DEX). In addition, **1d** and **1h** preserved collagen production (111.79 ± 1.56 for **1d**+DEX and 122.27 ± 1.56 for **1h**+DEX). In conclusion, this study suggests that the RSV derivatives **1d** and **1h** hold promise as potential antioxidant agents to counteract DEX-induced oxidative stress. These findings contribute to the development of novel therapeutic strategies for managing oxidative stress-related oral conditions.

## 1. Introduction

Oxidative stress, a condition caused by an imbalance between free radicals and antioxidants, has been linked to various age-related diseases and chronic conditions [1]. In detail, while small amounts of reactive oxygen species (ROS) play a role in cellular signaling, unchecked ROS production leads to oxidative stress, damaging cells, proteins, and DNA [2]. This excessive ROS production can harm delicate oral tissues like gums and even the surrounding tooth structure, potentially hindering post-surgical healing and exacerbating existing oral diseases [3]. Glucocorticoids have been utilized since their discovery to treat almost every autoimmune and chronic inflammatory illness due to their immunosuppressive and anti-inflammatory properties [4]. However, long-term therapies have side effects that may result in new disorders or may exacerbate the response to preexisting conditions such as osteoporosis [4,5]. For their potent anti-inflammatory effects, glucocorticoids are also widely used in dentistry to reduce swelling, pain, and discomfort after oral surgery procedures such as tooth extraction, wisdom tooth removal, and jaw surgery. In addition, glucocorticoids are employed to treat a variety of inflammatory oral diseases, such as recurrent aphthous stomatitis, oral lichen planus, and erythema multiforme. However, they show a lesser-known downside including an increased risk of infection, delayed wound healing, bone loss, and oral thrush [6,7]. These glucocorticoids-induced side effects could be linked to their potential to increase the production of ROS within oral tissues [8,9]. Therefore, supplementing with antioxidants alongside glucocorticoid therapy might offer a protective effect by reducing ROS production and promoting healing in the oral cavity [10].

**Figure 1:**
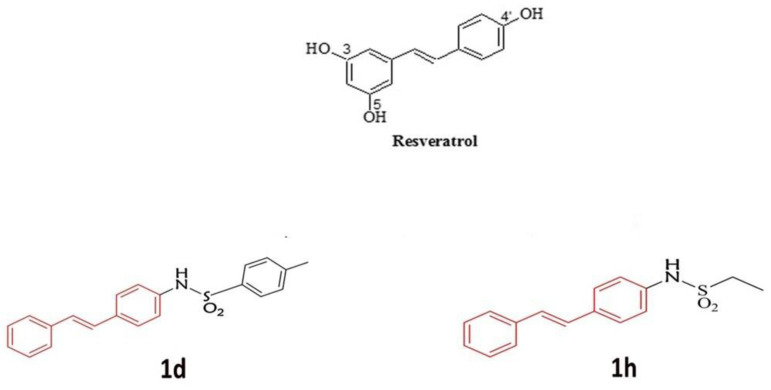
Structure of RSV and new sulfonamide derivatives of RSV **1d** with tosyl group and **1h** with an ethyl group.

Resveratrol (RSV) (Figure 1), a polyphenol detected in more than 70 plant species such as red vine, blueberry, and grapes, has garnered significant scientific interest due to its potential health benefits [11,12]. The main mechanism of action has been largely discussed [13,14]. Among the numerous pharmacological effects, the antioxidant effect is the most important [15]. This well-documented property has been measured in different in vitro assays [16,17]. RSV is a very effective scavenger of ROS as well as influencing the activity of numerous antioxidant enzymes responsible for maintaining the oxidation-reduction balance, such as superoxide dismutase (SOD), glutathione peroxidase (GPx), catalase (CAT), and heme oxygenase (HO-1). It reduces the activity of enzymes that play a dominant role in the production of ROS, such as xanthine oxidase (XO). RSV demonstrates an unusually strong ability to remove free radicals [18]. Its antioxidant activity is related to a variety of physiological and metabolic activities, and it acts in the prevention and treatment of numerous chronic diseases [19]. A recent systematic review has drawn attention to the beneficial effect of RSV on human microbiota, which plays a key role in maintaining an adequate immune response that can lead to different diseases when altered [20]. Another systematic review reported the positive effects of RSV on bone metabolism and its potential application as an adjuvant treatment for osteoporosis, bone tumors, and periodontitis [21].

Beyond its beneficial properties, the effectiveness of RSV is limited by its poor bioavailability [14]. The low bioavailability of RSV has encumbered its therapeutic application. To overcome this drawback, different strategies have been adopted [22]. Among them, the use of analogs with a better pharmacokinetic profile has been studied [23].

In our ongoing studies on the analogs of RSV [23,24,25,26,27,28], we focused on the synthesis of new structural RSV derivatives in which one phenolic functional group was linked to a lipophilic moiety by a sulfonamide linker [24]. Sulfonamide derivatives are frequently seen as structural motifs in medicinal chemistry and represent a large group of well-studied drugs that possess a wide range of biological activities [29]. The synthesis of sulfonamides is simple and provides a diversity of derivatives from a wide variety of amines and sulfonyl chlorides. The versatile structure of sulfonamides and the wide range of their activities have contributed to increased interest in repurposing old drugs. In this field, we investigated a series of RSV derivatives with better pharmacokinetic profiles [30]. In previous work, we studied the effects of a set of stilbene-containing sulfonamides [30]. These compounds can be considered derivatives of RSV with improved lipophilicity thanks to the presence of aromatic or aliphatic structural portions (Figure 1) [24]. They were synthesized in mild reaction conditions, using dry dichloromethane and triethylamine as a base, in an anhydrous atmosphere, and at room temperature [24]. To evaluate the cell metabolic activity, all these compounds were administered for 24 h and 72 h to HGFs in increasing concentrations (from 10 to 50 µM) using RSV as a reference at the same concentrations [30]. Among them, two compounds, namely **1d** (containing a tosyl group) and **1h** (containing an ethyl group) (Figure 1), positively and statistically increased cell viability with respect to control and RSV. They showed an upregulation of eNOS for endothelial cells, of COL1 for gingival fibroblasts, and of ALP for osteoblasts, at the lowest concentration [30]. They also showed favorable physicochemical properties, enzymatic and chemical stability, and good wound-healing properties [30]. Since structural analogy, derivatives can act in the same way as referent compounds with better results in scavenging ROS. Their increased activity may be due to their greater lipophilicity, as they have fewer hydrophilic functional groups (phenolic groups). To improve the knowledge of biochemical mechanisms underlying the obtained results, we considered exploring the antioxidant activity of these two RSV derivatives, **1d** and **1h**, in fibroblasts and osteoblasts under oxidative stress induced by dexamethasone. Fibroblasts and osteoblasts are essential cell types in connective tissues and bone, respectively. They play crucial roles in maintaining tissue structure, wound healing, and bone formation [31]. However, these cells are also susceptible to oxidative stress-induced damage, which can contribute to tissue degeneration and osteoporosis [32]. Since these assumptions, we hypothesized that the studied derivatives may exhibit enhanced antioxidant activity compared to RSV itself and could potentially protect these cells from oxidative stress-mediated damage. By exploring the effects of RSV derivatives on these cell types, we aim to contribute to the development of novel therapeutic strategies for oxidant-related conditions associated with oxidative stress.

## 2. Materials and Methods

### 2.1. Description of Derivatives of RSV

Compounds **1d** and **1h** were synthesized following reported procedures [24].

Compound **1d** is a derivative of RSV with stilbene-containing sulfonamides, and contains a tosyl group (Figure 1).

Compound **1h** is a derivative of RSV with stilbene-containing sulfonamides, and contains an ethyl group (Figure 1).

### 2.2. Cell Culture and Treatments

In this study, hGFs and hOBs were used. hGFs were purchased from ATCC (Manassas, VA, USA), while hOBs were isolated from mandible bone fragments of patients who underwent the surgical removal of lower third molars at the dental clinic of the G. D’Annunzio University. All patients signed an informed consent in accordance with the Declaration of Helsinki principles and according to the ethical standards of the Institutional Committee on Human Experimentation (reference number: BONEISTO N. 2210 July 2021). Immediately after sampling, each bone fragment underwent three enzymatic digestions at 37 °C for 20, 30, and 60 min utilizing a solution consisting of collagenase type 1A (Sigma-Aldrich, St. Louis, MO, USA) and trypsin-EDTA 0.25% (Sigma-Aldrich) dissolved in Dulbecco’s Modified Eagle’s medium (DMEM, Corning, New York, NY, USA) at 10% fetal bovine serum (FBS, Gibco-Life Technologies, Monza, Italy). The solution obtained from the enzymatic digestion was centrifuged at 1200 rpm for 10 min. Then, the pellet obtained was transferred into a T25 culture flask with low-glucose (1 g/L) DMEM supplemented with 10% FBS, 1% antibiotics (100 μg/mL^−1^ streptomycin and 100 IU/mL^−1^ penicillin), and 1% L-glutamine to promote a final spontaneous migration of the cells. The isolated hOBs were cultured at 5% CO_2_ and 37 °C to achieve their confluence to be used between the 3rd and the 5th passage upon the characterization by cytometric analysis. Following 10 days of culture, the bone fragments were removed.

[NO_PRINTED_FORM]

hGFs and hOBs were cultured using low-glucose DMEM (1 g/L) (Corning, New York, NY, USA) supplemented with 10% fetal bovine serum (FBS) (SIAL, Rome, Italy), 1% penicillin and streptomycin, and 1% of L-glutamine (Corning) at 37 °C and 5% CO_2_.

The following experimental groups were distinguished:

- (CTRL) cells were treated with 0.1% of DMSO;
- (RSV) cells were treated with 5 µM RSV;
- (**1d**) cells were treated with 5 µM **1d** derivatives;
- (1h) cells were treated with 5 µM **1h** derivatives;
- (DEX) cells were treated with 200 µM of dexamethasone (DEX; Sigma Aldrich, St. Louis, MO, USA) for 30 min to induce oxidative stress. This dose was chosen among different concentrations based on their potential to induce the highest level of ROS (Figure S1 in the Supplementary Materials);
- (RSV+DEX) cells were treated with 200 µM of DEX. After treatment with DEX, the medium containing DEX was removed, and cells were treated with 5 µM RSV;
- (**1d**+DEX) cells were treated with 200 µM of DEX. After treatment with DEX, the medium containing DEX was removed, and cells were treated with 5 µM **1d**;
- (1h+DEX) cells were treated with 200 µM of DEX. After treatment with DEX, the medium containing DEX was removed, and cells were treated with 5 µM **1h**.

### 2.3. Cell Viability

Cell viability was assessed after 48 and 72 h using CellTiter96 assay (MTS) (Promega, Madison, WI, USA), as previously described [33]. In this experiment, 1 × 10^4^ cells were seeded in each well of a 96-well plate and exposed to treatment with 200 μM of DEX for 30 min and then with RSV, **1d**, and **1h**. Afterward, 10 μL of MTS solution was added, and the plate was incubated for 3 h at 37 °C and 5% CO_2_. A microplate reader (Synergy H1 Hybrid BioTek Instruments, Santa Clara, CA, USA) then measured the absorbance at 490 nm, which is proportional to the number of viable cells. Cell viability was expressed as a percentage (%).

### 2.4. CSLM

Cells were cultured on 8-well culture slides at a density of 1.3 × 10^4^ cells/well. Then, they were treated for 72 h. After fixation with 4% paraformaldehyde in 0.1 M phosphate-buffered saline (PBS), the cells were washed three times in PBS and permeabilized with 0.1% Triton X-100 in PBS for 5–6 min. The cytoskeletal actin and the nuclei were stained, respectively, with rhodamine-phalloidin (Invitrogen) and DAPI (4′,6-diamidino-2-phenylindole dihydrochloride; Sigma), both prepared at a 1:1000 dilution in PBS and incubated for 1 h at 37 °C. The images were acquired using the Zeiss LSM800 confocal microscope (Carl Zeiss, Jena, Germany).

### 2.5. ROS Levels

hGFs and hOBs cells (1 × 10^4^ cells/well) were seeded on a 96-well plate in order to examine how much ROS accumulated after treatment. After a day, the cells were exposed to treatment with 200 μM of DEX for 30 min and then with RSV, **1d**, and **1h**. Following the protocol, a kit (Abcam, Cat No. ab186027, Cambridge, UK) was used to measure ROS levels at 0, 1, and 4 h after treatment. Briefly, DCFH-DA (working solution) at 10 μM was added to each well and incubated for 30 min. A microplate spectrofluorometer (Synergy H1 Hybrid BioTek Instruments) was used to measure the intensity of the fluorescence at λ ex/em 520/605 nm.

### 2.6. SOD Activity

In a 96-well plate, 2 × 10^4^ cells/well were seeded and grown for 24 h in complete low-glucose DMEM medium. Then, the cells were exposed to treatment with 200 μM of DEX for 30 min and then with RSV, **1d**, and **1h**. SOD activity was measured after 0, 1, and 4 h from treatment, using a commercial SOD assay kit at 450 nm (Abcam, Cat No. ab65354) following the manufacturer’s instructions.

### 2.7. Gene Expression

mRNA expression levels of oxidative stress markers (SOD1, GSH, CAT, OH-1, and NRF2) were evaluated at 72 h using RT-qPCR. Total RNA was isolated using 1 mL of Trifast reagent (EuroClone, Pero, Italy) and subsequent passages in chloroform, isopropanol, and ethanol. The RNA integrity, purity, and concentration were then analyzed using a Nanophotometer NP80 spectrophotometer (Implen NanoPhotometer, Westlake Village, CA, USA). Next, the GoTaq^®^ 2 Step RT-qPCR Kit (Promega, Madison, WI, USA) was used to convert the RNA into complementary DNA (cDNA). SYBR Green was used for RT-qPCR, following the manufacturer’s instructions. Targets and housekeeping genes were amplified in a volume of 10 μL containing 1 μL of cDNA template, 0.2 μL of primers mixture, and 5 μL of GoTaq^®^ 2-Step RT-qPCR system (Promega). A Quant Studio 7 Pro Real-Time PCR System (ThermoFisher, Waltham, MA, USA) was used to measure gene expression. Glyceraldehyde-3-Phosphate Dehydrogenase (GAPDH) was used as a housekeeping gene for hGFs, while beta-actin (β-ACT) for hOBs. The 2^−ΔΔCt^ method was employed to normalize the results. The primer sequences used are listed in Table S1 in the Supplementary Materials.

### 2.8. Picro-Sirius Red Staining and Spectrophotometric Analysis

hGFs and hOBs were seeded onto 24-well plates at a density of 5 × 10^4^ cells/well and treated after 24 h. After seven days, the cells were fixed with a 2.5% glutaraldehyde solution for 2 h. Subsequently, they were incubated with 1 mg/mL Ditect red staining solution (Sigma Aldrich) at room temperature for 1 h. Cells then underwent three washes with a 0.1% acetic acid solution. Images were captured using a Leica stereomicroscope at 25× magnification. To quantify collagen content, Picro-Sirius red was eluted with 0.1 N sodium hydroxide for one hour, followed by spectrophotometric analysis at 540 nm wavelength using a microplate reader (Synergy H1 Hybrid, BioTek Instruments).

### 2.9. Statistical Analysis

GraphPad 8.0.2.263 software (GraphPad, San Diego, CA, USA) was used for statistical analysis. One-way ANOVA was employed, followed by a post hoc Tukey’s multiple comparisons test to assess significant differences between groups. A *p*-value of less than 0.05 was considered statistically significant. The RSV, **1d**, and **1h** experimental groups were compared to CTRL. The RSV+DEX, **1d**+DEX, and **1h**+DEX experimental groups were compared to the DEX group.

## 3. Results

### 3.1. Influence of **1d** and **1h** in Combination with Dexamethasone on Cell Viability

**Figure 2:**
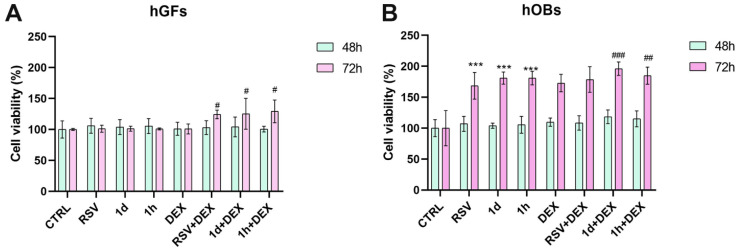
Cell viability of hGFs (**A**) and hOBs (**B**) after treatment with 5 µM RSV and **1d** and **1h** alone or in combination with 200 µM DEX at 48 and 72 h. *** *p* < 0.0001 vs. CTRL; # *p* < 0.05 vs. DEX; ## *p* < 0.001 vs. DEX; ### *p* < 0.0001 vs. DEX.

Figure 2A shows the impact of treatments with RSV and RSV derivatives in combination with dexamethasone (DEX) on the viability of hGFs. RSV treatment alone did not significantly affect cell viability compared to CTRL at either 48 or 72 h. **1d** and **1h** showed a similar effect on cell growth with respect to RSV and CTRL at both time points. DEX also appeared to have no effect on hGF viability at 48 and 72 h. Interestingly, the combination of RSV and DEX (RSV+DEX) resulted in higher cell viability compared to RSV alone at both 48 and 72 h. Compared to **1d** and **1h** alone, **1d**+DEX and **1h**+DEX showed increased viability. Furthermore, statistically significant enhancements in cell viability were observed for RSV+DEX, **1d**+DEX, and **1h**+DEX compared to DEX treatment (*p* < 0.05). The viability of hOBs is shown in Figure 2B. At 48 h, RSV, **1d**, and **1h** treatments alone did not significantly influence hOBs viability compared to CTRL. However, a slight increase in viability was observed when cells were treated with a combination of RSV, **1d**, or **1h** with DEX. At 72 h, a statistically significant rise in hOBs viability was observed after treatment with RSV, **1d**, or **1h** compared to the control group (*p* < 0.0001). Interestingly, the addition of DEX to these treatments (RSV+DEX, **1d**+DEX, and **1h**+DEX) further enhanced cell growth in a statistically significant manner. This is evidenced by the viability percentages, which were 178.63 ± 20.68, 196.17 ± 10.92, and 184.80 ± 13.87 for RSV+DEX, **1d**+DEX, and **1h**+DEX, respectively.

### 3.2. Influence of **1d** and **1h** in Combination with Dexamethasone on Morphology

**Figure 3:**
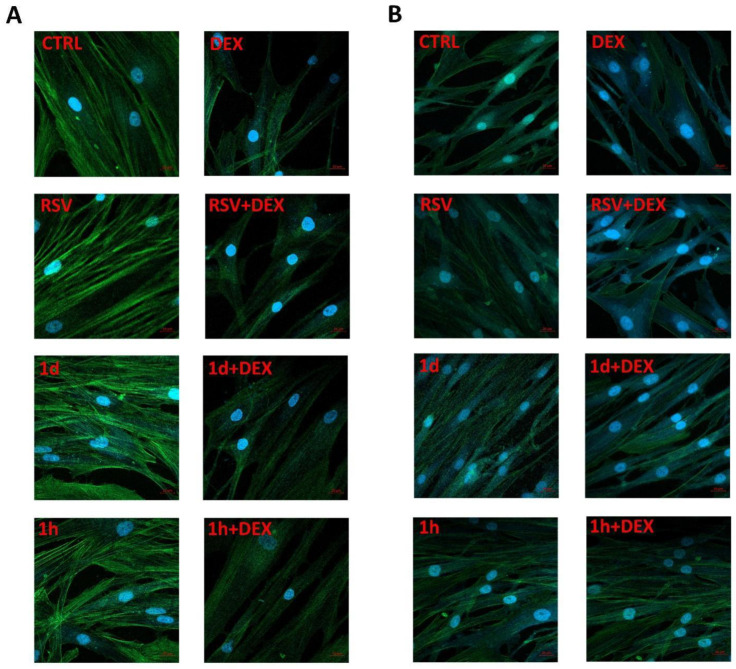
Immunofluorescence images of hGFs (**A**) and hOBs (**B**) morphology, without treatments and after treatment with 5 µM RSV and **1d** and **1h** alone or in combination with 200 µM DEX. Cells were stained with rhodamine-phalloidin (green) and DAPI (blue).

Figure 3 shows images of cells, with and without treatments, produced by immunofluorescence assay. The strength of the green intracellular fluorescence corresponds to cytoskeletal filaments while the blue one corresponds to nuclei. In hGFs, any difference in green and blue fluorescence was detected between the RSV, **1d**, and **1h** groups compared to the CTRL group. The fluorescence intensity decreased in the DEX-treated hGFs while RSV, **1d**, and **1h** (5 µM) increased green fluorescence. The morphology of hGFs appeared less tapered in the presence of DEX. In hOBs, a difference in green and blue fluorescence was detected between the RSV, **1d**, and **1h** groups compared to the CTRL group indicating an increase in cell number. The fluorescence intensity decreased in the DEX-treated hOBs while RSV, **1d**, and **1h** (5 µM) remarkably increased blue fluorescence. The morphology of hOBs appeared less tapered in the presence of DEX. **1h** seemed to favor the spindle-shaped morphology of DEX-treated hOBs.

### 3.3. Influence of **1d** and **1h** in Combination with Dexamethasone on ROS Levels

**Figure 4:**
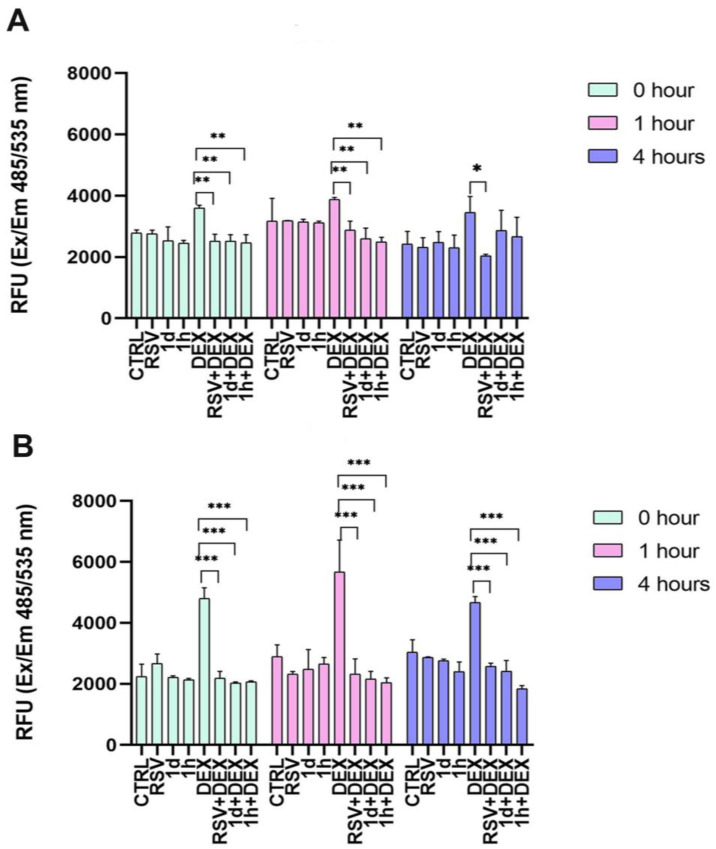
ROS production induced by treatment with 5 µM RSV and **1d** and **1h** alone or in combination with 200 µM DEX at 0, 1, and 4 h in hGFs (**A**) and hOBs (**B**). RFU: relative fluorescence unit. * *p* < 0.05 vs. DEX; ** *p* < 0.001 vs. DEX; *** *p* < 0.0001 vs. DEX.

The impact of the treatments with RSV and RSV derivatives combined with DEX on ROS levels in hGFs (Figure 4A) and hOBs (Figure 4B) were evaluated at 0, 1, and 4 h. In hGFs, treatment with RSV, **1d**, and **1h** alone did not significantly increase ROS production compared to CTRL at any time point (Figure 3A). DEX treatment alone elevated ROS levels. In detail, at 0, 1, and 4 h, the measured relative fluorescence units (RFU) were 3600.50 ± 88.39, 3873.50 ± 67.18, and 3453.00 ± 520.43, respectively. Interestingly, the addition of RSV or its derivatives to the culture with DEX (RSV+DEX, **1d**+DEX, **1h**+DEX) statistically reduced ROS levels at 0 and 1 h compared to DEX alone. At 4 h, the RSV+DEX combination showed a statistically significant decrease in ROS levels (2043.67 ± 47.12). In hOBs, exposure to RSV, **1d**, and **1h** alone did not significantly affect ROS production at any time point (Figure 4B). DEX treatment alone increased ROS levels, reaching its peak at 1 h, with a measured value of 5660 ± 1054.83 RFU. Interestingly, subsequent exposure to RSV, **1d**, or **1h** (RSV+DEX, **1d**+DEX, **1h**+DEX) significantly reduced ROS levels 2325.33 ± 491.75, 2153.67 ± 254.96 and 2040.00 ± 154.28, respectively. These reductions brought ROS levels down to values comparable to CTRL (2887.33 ± 389.36).

### 3.4. Influence of **1d** and **1h** in Combination with Dexamethasone on SOD Activity

**Figure 5:**
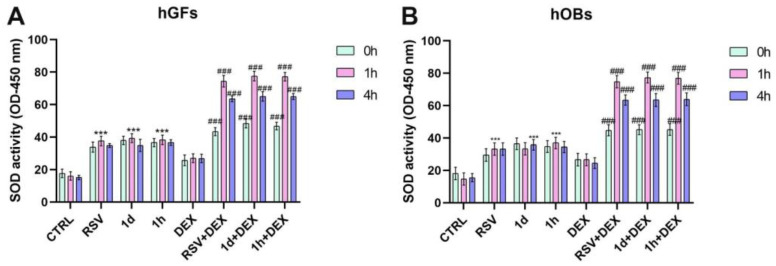
Evaluation of SOD activity after treatment with 5 µM RSV and **1d** and **1h** alone or in combination with 200 µM DEX at 0, 1, and 4 h in hGFs (**A**) and hOBs (**B**). OD: optical density. *** *p* < 0.0001 vs. CTRL; ### *p* < 0.0001 vs. DEX.

The impact of RSV and RSV derivatives combined with DEX on SOD activity in hGFs (Figure 5A) and hOBs (Figure 5B) was evaluated. SOD activity was measured at 0, 1, and 4 h after treatments. In hGFs, SOD activity resulted statistically increased in RSV, **1d**, and **1h** groups with respect to CTRL (***p < 0.0001) (Figure 5A). The treatment with DEX induced a slight increase in SOD activity compared to CTRL. In addition, the lowest SOD activity was observed at 0 h after co-treatment with **1d** and **1h**, while the highest activity was observed at 1 h. These results were statistically significant compared to DEX (### *p* < 0.0001). Similar to hGFs, treatment with RSV, **1d**, or **1h** alone statistically increased SOD activity in hOBs compared to CTRL (*** *p* < 0.0001) (Figure 5B). The activity ranged between 29.46 and 36.93 in terms of optical density (OD). DEX treatment alone resulted in a slight increase in SOD activity. All combination treatments (RSV+DEX, **1d**+DEX, and **1h**+DEX) significantly elevated SOD activity compared to the DEX group (26.70 ± 3.87, 26.60 ± 3.62, and 24.53 ± 3.29 at 0, 1 and 4 h, respectively). In particular, the values of RSV+DEX were 44.80 ± 3.42, 74.83 ± 3.73, and 63.47 ± 3.19 at 0, 1, and 4 h, respectively. The values of **1d**+DEX resulted in 45.17 ± 3.08, 77.33 ± 3.34, and 63.50 ± 3.98 at 0, 1 and 4 h, respectively. The values of **1h**+DEX were 45.27 ± 3.64, 76.87 ± 3.59, and 63.97 ± 3.95 at 0, 1 and 4 h, respectively.

### 3.5. Influence of **1d** and **1h** in Combination with Dexamethasone on Antioxidant Gene Expression

**Figure 6:**
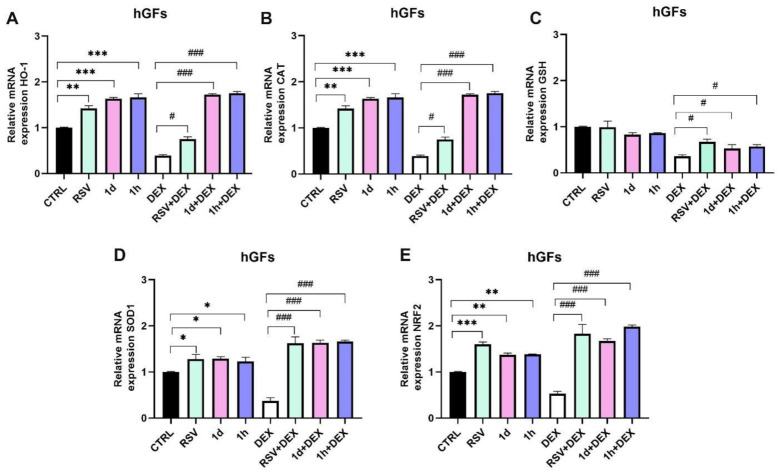
Gene expression of antioxidant genes after treatment with 5 µM RSV and RSV derivatives (**1d** and **1h**) alone or in combination with 200 µM DEX in hGFs. HO-1: Heme oxgenase-1 (**A**); CAT: Catalase (**B**); GSH: Glutathione (**C**); SOD1: Superoxide dismutase 1 (**D**); NRF2: nuclear factor erythroid 2–related factor 2 (**E**). * *p* < 0.05 vs. CTRL; ** *p* < 0.001 vs. CTRL; *** *p* < 0.0001 vs. CTRL; # *p* < 0.05 vs. DEX; ### *p* < 0.0001 vs. DEX.

**Figure 7:**
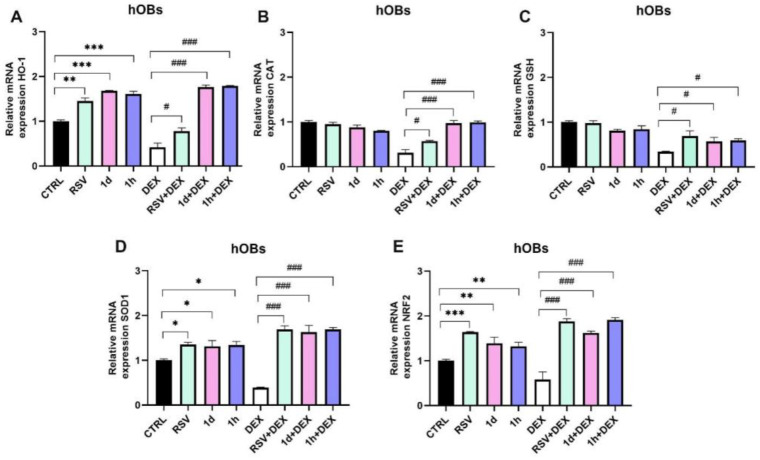
Gene expression of antioxidant genes after treatment with 5 µM RSV and RSV derivatives (**1d** and **1h**) alone or in combination with 200 µM DEX in hOBs. HO-1: Heme oxgenase-1 (**A**); CAT: Catalase (**B**); GSH: Glutathione (**C**); SOD1: Superoxide dismutase 1 (**D**); NRF2: nuclear factor erythroid 2–related factor 2 (**E**). * *p* < 0.05 vs. CTRL; ** *p* < 0.001 vs. CTRL; *** *p* < 0.0001 vs. CTRL; # *p* < 0.05 vs. DEX; ### *p* < 0.0001 vs. DEX.

Figure 6 and Figure 7 show the expression of antioxidant genes in hGFs and hOBs, respectively. Heme oxygenase-1 (HO-1) and catalase (CAT) displayed similar trends (Figure 6A,B). RSV treatment significantly increased HO-1 (1.42 ± 0.06) and CAT (1.47 ± 0.06) mRNA expression compared to the control group (** *p* < 0.001). Interestingly, **1d** and **1h** treatments also significantly increased HO-1 mRNA expression compared to the control 1.63 ± 0.03 and 1.66 ± 0.08, respectively, even more than RSV alone. DEX treatment downregulated HO-1 (0.39 ± 0.02) and CAT (0.41 ± 0.03), but this effect was reversed by the addition of RSV, **1d**, or **1h**. In detail, RSV significantly improved HO-1 (0.75 ± 0.05) and CAT (0.77 ± 0.04) mRNA expression, RSV treatment alone did not affect glutathione (GSH) expression, while **1d** and **1h** treatments caused a slight decrease compared to CTRL (Figure 6C). DEX downregulated GSH (0.36 ± 0.03), but its expression was statistically increased in the RSV+DEX (0.67 ± 0.06), **1d**+DEX (0.53 ± 0.08), and **1h**+DEX (0.57 ± 0.04) groups compared to DEX alone. However, **1d** and **1h** in combination with DEX showed a small decrease in expression compared to RSV+DEX.

Superoxide dismutase 1 (SOD1) mRNA expression was promoted by RSV, **1d**, and **1h** treatments compared to CTRL (Figure 6D). DEX reduced SOD1 expression. The combination of RSV and DEX (1.62 ± 0.14), along with the combination of derivatives **1d** (1.63 ± 0.14) and **1h** (1.66 ± 0.03) and DEX, reversed this effect, leading to a statistically significant increase in SOD1 expression compared to DEX alone (0.37 ± 0.07). Nuclear factor erythroid 2-related factor 2 (NRF2) mRNA expression was significantly increased in the RSV (1.60 ± 0.05), **1d** (1.37 ± 0.04), and **1h** (1.38 ± 0.01) groups compared to the control (Figure 6E). As observed with other genes, DEX downregulated NRF2 expression (0.53 ± 0.05). However, co-treatment with RSV and DEX (1.83 ± 0.20), **1d** and DEX (1.67 ± 0.05), or **1h** and DEX (1.98 ± 0.04) significantly upregulated NRF2 expression (### *p* < 0.0001).

In hOBs, RSV (1.45 ± 0.07), **1d** (1.68 ± 0.01), and **1h** (1.61 ± 0.06) treatments significantly increased HO-1 mRNA expression compared to the control group (Figure 7A). DEX treatment downregulated HO-1 expression (0.42 ± 0.09), but co-treatment with RSV (0.78 ± 0.07), **1d** (1.76 ± 0.05), or **1h** (1.79 ± 0.01) reversed this effect. CAT expression levels were comparable among the RSV, **1d**, **1h**, and control groups (Figure 7B). Co-treatment of RSV+DEX (0.57 ± 0.02), **1d**+DEX (0.97 ± 0.06), and **1h**+DEX (0.99 ± 0.03) statistically increased CAT expression compared to DEX alone (0.31 ± 0.07). Similar to hGFs, RSV alone did not affect GSH expression in hOBs, while **1d** and **1h** treatments caused a slight decrease (Figure 7C). DEX treatment lowered GSH expression (0.34 ± 0.01), but co-treatments with RSV+DEX (0.69 ± 0.12), **1d**+DEX (0.57 ± 0.09), or **1h** +DEX (0.59 ± 0.04) increased its expression. Treatments with RSV, **1d**, and **1h** slightly increased SOD1 mRNA levels (Figure 7D). DEX treatment decreased SOD1 expression (0.39 ± 0.01). Interestingly, adding RSV+DEX (1.69 ± 0.08), **1d**+DEX (1.63 ± 0.15), and **1h**+DEX (1.69 ± 0.04) reversed this decrease, leading to a statistically significant increase in SOD1 expression compared to DEX alone (### *p* < 0.0001). As observed in hGFs, RSV (1.64 ± 0.01), **1d** (1.39 ± 0.14), and **1h** (1.32 ± 0.09) treatments significantly increased NRF2 mRNA expression compared to the control group (Figure 7E). DEX downregulated NRF2 expression (0.58 ± 0.17), similar to other genes. However, the combination of RSV+DEX (1.88 ± 0.06), **1d**+DEX (1.62 ± 0.04), or **1h**+DEX (1.91 ± 0.05) with DEX reversed this effect, resulting in a statistically significant improvement in NRF2 expression (### *p* < 0.0001).

### 3.6. Influence of **1d** and **1h** in Combination with Dexamethasone on Collagen Production

**Figure 8:**
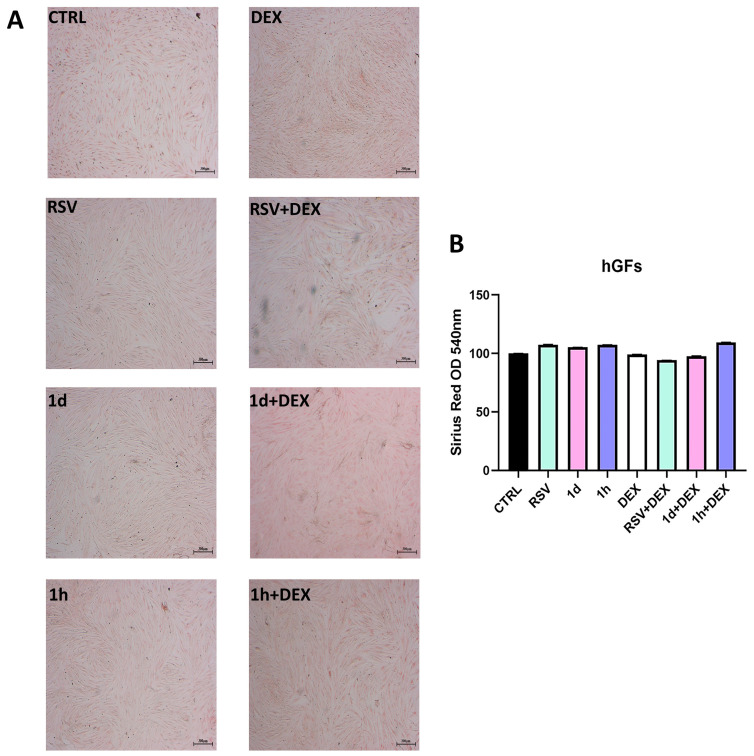
Effects of treatment with 5 µM RSV, **1d** and **1h** combined and not with 200 µM DEX on collagen production in hGFs. Picrosirius red staining observation (**A**) was quantified by spectrophotometric analysis at 540 nm (**B**). Magnification: 25×; scale bar 300 μm.

**Figure 9:**
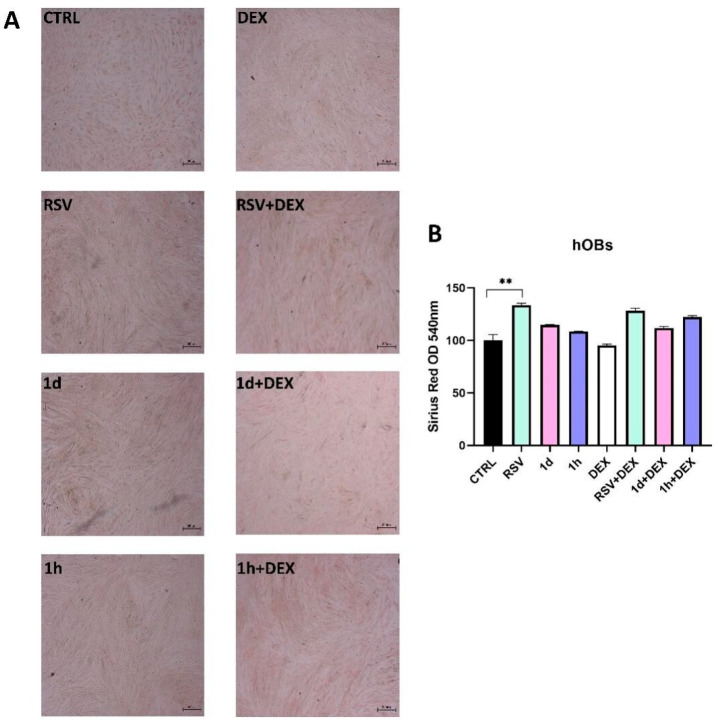
Effects of treatment with 5 µM RSV, **1d** and **1h** combined and not with 200 µM DEX on collagen production in hOBs. Picrosirius red staining observation (**A**), magnification: 25×; scale bar 300 μm. Quantification by spectrophotometric analysis at 540 nm, ** *p* < 0.001 vs. CTRL (**B**).

Figure 8 and Figure 9 show the evaluation of collagen production, assessed qualitatively by Picro-Sirius Red Staining and quantitatively by spectrophotometric analysis. In hGFs, all treatments did not appear to influence collagen production (Figure 8). Specifically, a similar intensity of red coloration was observed across all treatment groups (Figure 8A). This qualitative analysis was further confirmed by spectrophotometric measurements (Figure 8B).

Similar to hGFs, the qualitative analysis of hOBs revealed comparable collagen production across all conditions (Figure 9A). RSV, **1d**, and **1h** treatments stimulated collagen production compared to the control. However, only RSV reached statistical significance (133.62 ± 1.84%). Interestingly, co-treating RSV, **1d**, or **1h** with DEX resulted in a slight increase in collagen production compared to DEX alone (Figure 9B).

## 4. Discussion

Prolonged administration of glucocorticoids results in fibroblast activity suppression, loss of connective tissue and collagen, as well as a reduction in angiogenesis and re-epithelization [34]. One of the most accredited mechanisms of glucocorticoids-induced side effects is oxidative stress [35]. On the other hand, antioxidant supplementation can mitigate the damaging effect of induced oxidative stress. To this end, in this study, RSV and two derivatives were administrated at the concentration of 5 µM to DEX-treated cells, such as hGFs and hOBs, which mainly are involved in the physiology of oral tissues. The viability of hGFs was not affected by the dispensation of 200 µM of DEX for 30 min, nor by supplementation with RSV, **1d,** and **1h** alone. Meanwhile, treatment with RSV as well as its derivatives had beneficial effects on the viability of oral hOBs after 72 h. Interestingly, the addition of RSV or derivatives to both hGFs and hOBs pre-treated with DEX had a stimulatory effect on cell proliferation. Like the cell viability results, the effect was higher for derivatives than for RSV. Images of cell morphology detected by immunofluorescence were in line with the results of viability. Images from the confocal microscope showed that cells in the presence of DEX displayed a less tapered morphology, while the addition of RSV derivatives favors the typical spindle-shaped morphology of these cells. To investigate whether derivatives **1d** and **1h** could mimic RSV as antioxidative agents, the DEX-induced intracellular ROS levels were determined by using the H2DCFDA marker. The results demonstrated that hOBs displayed higher levels of DEX-induced ROS than hGFs, indicating a greater sensitivity to this glucocorticoid. Dexamethasone can probably cause higher ROS levels in osteoblasts than fibroblasts because osteoblasts are more specialized and metabolically active, making them more sensitive to dexamethasone’s disruptive effects on cellular processes. There is growing evidence that shows how DEX-induced oxidative stress contributes to osteoporosis [32,36,37]. Several in vitro studies reported that DEX may cause osteoblast dysfunction with consequent bone loss [38,39]. Liu S. et al. showed that DEX-treated osteoblasts exhibited lower mRNA levels of osteogenic genes, such as Runx2, osterix, bone morphogenetic protein-2, and osteocalcin. Furthermore, treatment with DEX was associated with diminished ALP activity in osteoblasts, as well as fewer calcium deposits, compared to untreated ones [39].

In this study, the protective effects of RSV and its derivatives against DEX-induced ROS were time-dependent in hGFs. The intracellular ROS inhibition by derivatives **1d** and **1h** was significantly higher than that of their parent compound RSV 1 h post treatments, but it was lower after 4 h. Furthermore, there was no significant difference between **1d** and **1h**. In hOBs, derivatives **1d** and **1h** both reduced DEX-induced ROS levels more than RSV and in a time-dependent manner, with a great effect 4 h after treatment with **1h** compound. Oxidative stress takes place when the scavenging action of intracellular antioxidants and the production of highly reactive oxygen species get out of balance. The physiological level of ROS is essential for the maintenance of normal cellular function, while excessive production of ROS leads to mitochondrial damage and cell injury [40]. In our study, cell viability assays showed that treatment with DEX, RSV, and its derivatives was associated with increased proliferative activity in cells, mainly in hOBs. On the other hand, high levels of DEX-induced ROS were also observed. Thus, we hypothesized that RSV and compounds **1d** and **1h** were able to stimulate the scavenging ability of antioxidant enzymes such as SOD. In our study, hGFs and hOBs showed the same trend in SOD activity. Compared to untreated cells, RSV and its derivatives had a significant stimulatory effect on the activity of this enzyme. In contrast, when cells were subjected to DEX, the increment of SOD activity was slightly increased, indicating that cells trigger a physiological reaction to the stressor glucocorticoid. A significant increment was observed when DEX-treated cells also received RSV, **1d**, and **1h** compounds, with the highest levels of SOD activity at 1 h post treatments. It is well known that the SOD enzyme counteracts superoxide anion (O^2−^) radicals, but ROS also include hydroxyl radicals (OH^−^) and hydrogen peroxide (H_2_O_2_), which can damage many cell components such as DNA, proteins, and lipids [40]. Since RSV and its derivatives were able to decrease the DEX-induced ROS, we hypothesized that antioxidant systems other than SOD were involved. In the present study, the expression of key antioxidant genes was investigated. The NRF2 expression was higher in hGFs and hOBs which received RSV and its derivatives compared to the control. Again, in DEX-treated cells, NRF2 was decreased and inversely increased in the RSV and derivatives groups. NRF2 acts as a transcription factor that regulates the intracellular redox balance and the antioxidants in the cell and regulates inflammation, senescence, and ROS [41]. It is well known that RSV triggers NRF2 signaling activation [42]. In our study, derivatives **1d** and **1h** showed similar effects to RSV, indicating that they could replace RSV as antioxidative agents in triggering NRF2. Upon exposure of cells to oxidative stress, NRF2 translocates into the nucleus to bind to antioxidant-responsive elements in genes encoding antioxidant enzymes, such as HO-1, SOD1, and SOD2 [41]. The upregulation in the expression of HO-1 was induced by RSV and its derivatives without significant differences among them. Upon exposure of cells to DEX, the expression of these genes dramatically decreased, and inversely increased when RSV or compounds **1d** and **1h** were added. It was observed that **1d** and **1h** provoked a higher upregulation of HO-1 mRNA in DEX-treated cells than RSV, indicating that the derivatives had a significantly higher radical scavenging ability than RSV. During normal cellular activities, SOD catalyzes superoxide anion radicals to hydrogen peroxide (H_2_O_2_), while CAT and GSH convert H_2_O_2_ to water (H_2_O) and oxygen (O_2_) [43]. In our experiments, SOD1 mRNA levels were significantly stimulated by RSV and by **1d** and **1h** compounds in hGFs compared to the control. It has been also determined that DEX reduces SOD1, GSH, and CAT expression in both hGFs and hOBs. This condition was reverted by adding RSV and its derivatives. In particular, it was observed that **1d** and **1h** stimulated a higher upregulation of CAT mRNA in DEX-treated cells than in RSV. In recent years, in vitro and in vivo studies focused on the biological properties of resveratrol which primarily include antioxidant and anti-inflammatory activities, anti-platelet aggregation effects, anti-atherogenic properties, estrogen-like growth-promoting effects, and immunomodulation [12]. However, studies have also demonstrated that resveratrol exhibits pro-oxidant properties, depending on the concentration and the cell type. Indeed, in different cell types, such as a fibroblast cell line and tumor human cells, RSV was found to exert its cytotoxic action at doses higher than 20 µM [44,45,46]. The underlying mechanism of pro-oxidant action seems linked to oxidative breakage of cellular DNA, in particular in the presence of transition metal ions such as copper [47]. Thus, the dualistic behavior of RSV poses it as an active redox molecule [48]. Martins L.A.M et al. evaluated the effects of RSV at concentrations of 0.1, 1, 10, and 50 μM on murine hepatic stellate (GRX) cells viability and oxidative status. While low doses of RSV did not affect GRX viability, the higher dose (50 μM) significantly provoked cell death via induction of oxidative stress. Interestingly, the analysis of SOD and CAT activity revealed an opposite effect of RSV depending on its dose. The activity of these antioxidant enzymes was promoted when cells were treated with 0.1–10 μM of RSV, while cells treated with 0.1–10 μM of RSV presented a decrease in these enzyme activities [49]. Thus, this study well describes the dual effects of a molecule like RSV and how its effects can depend on applied concentrations. In our study, the increasing activity of antioxidant assets, especially the SOD enzyme, should be considered as an antioxidant effect and not a prooxidant consequence, because RSV and its derivatives showed a beneficial effect on cell viability by promoting cell proliferation. Among the several extra cellular matrix (ECM) proteins, collagen type I is extensively expressed, and it represents a marker of bone and soft tissue turnover [12]. Collagen synthesis by hGFs was not affected by treatment with DEX as well as by the supplementation of RSV, **1d,** and **1h**, whereas the ability of hOBs to deposit collagen had beneficial effects by treatments with RSV. Our results are in line with data from the literature, even if the effects of RSV on Collagen I synthesis by osteoblasts are controversial [50,51,52]. In this research work, the lipophilicity of the RSV derivatives was varied by design through controlling the balance of hydrophobic features to polar and ionic features [53]. Their increased lipophilicity, as reported previously [30], can result in increased binding due to non-specific interactions with the biological targets, and to an increased ability to cross cell membranes and reach targets more effectively. The detailed study of this aspect will be the subject of future work.

## 5. Conclusions

RSV, a natural compound known especially for its antioxidant properties, has been shown to possess remarkable health benefits. However, the pharmacokinetic problems that result in its poor bioavailability limit its therapeutic application. For this reason, the structural modification of RSV has received more particular attention, and researchers developed many structural derivatives.

Starting from previous results, we aim to focus on the potential antioxidant activities of two homemade RSV derivatives. In this study, compounds **1d** and **1h** were assayed in gingival fibroblasts and oral osteoblasts, essential cell types in connective tissues and bone, respectively.

Our results highlighted their protective effects against DEX-induced oxidative stress, by regulating antioxidant parameters such as Nrf2, SOD, GSH, HO-1, and CAT, better than RSV. This fact could be attributed to the loss of phenolic functions of RSV and the combination of the sulfonamide moiety with increasing lipophilicity that promotes intracellular activity.

The obtained data led us to consider **1d** and **1h** valid starting points for the design of new compounds with potential activity against oxidative stress-related oral damage.

## Data Availability

Data are contained within the article and Supplementary Materials.

## Supplementary Materials

The following supporting information can be downloaded at: https://www.mdpi.com/article/10.3390/metabo14070350/s1, Figure S1. Reactive oxygen Species (ROS) measurement; Table S1. Primer sequences.
