# Supplementary material for: The Application of Resveratrol Derivatives in Oral Cells Reduces the Oxidative Stress Induced by Glucocorticoids
Source: Metabolites. 2024 Jun 22;14(7):350. doi: 10.3390/metabo14070350 (PMC11279245; doi:10.3390/metabo14070350)
Supplement: Supplementary file 1 [file metabolites-14-00350-s001.zip › metabolites-3054289-supplementary.pdf]

Supplementary Materials

- **Figure S1 Reactive oxygen Species (ROS) measurement**
- **Table S1 Primer sequences**

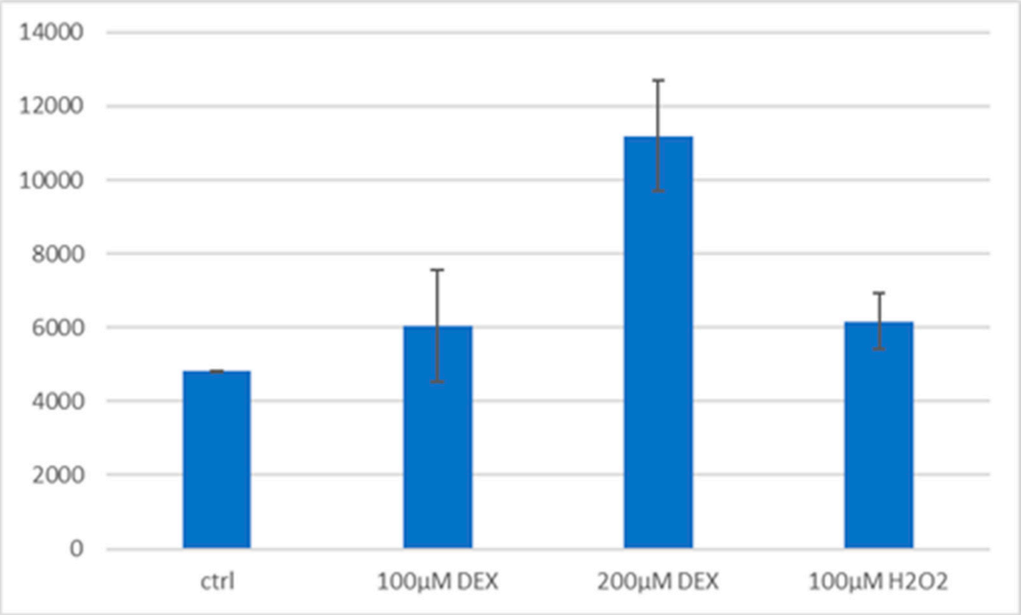

Figure S1. Dexamethasone (DEX) dose used in the experiments has been chosen on the basis of Reactive oxygen Species (ROS) production comparing the levels of generated ROS induced by DEX with that induced by H_2O_2. ROS levels were expressed in relative fluorescence unit (RFU).

Table S1. Primer sequences

Gene	Forward Primer (5'-3')	Reverse Primer (3'-5')
**CAT**	GCGGGCATTCTATGTGAACG	CGTAGTCAGGGTGGACCTCA
**HO-1**	CAGAAAGTTCAGACTTGGCTAGG	TCATTGTCATAGTGGTGGGAAC
**GSH**	TGCCTCCAAAGTATGTCAATCA	ACACGGTCCACAGCTCATC
**NRF2**	AGCCCATGTTGTAGCAAACC	TCTCAGCTCCACGCCATT
**GAPDH**	GGAGGGATCTCGCATTTCTT	ACGGGAAGCTTGTCATCAAT
**β-ACT**	CCAGAGGCGTACAGGGATAG	GAGAAGATGACCCAGGACTCTC
